# Unveiling Risk Profiles: A Latent Profile Analysis of 21st‐Century Skills, Resistance to Change, and Cognitive Flexibility

**DOI:** 10.1002/brb3.70167

**Published:** 2025-01-07

**Authors:** Muhammed Celal Uras, Suat Kaya, Alican Kaya, Murat Yildirim

**Affiliations:** ^1^ Department of Mathematics Education, Faculty of Education Agri Ibrahim Cecen University Ağrı Turkey; ^2^ Educational Sciences, Faculty of Education Agri Ibrahim Cecen University Ağrı Turkey; ^3^ Department of Guidance and Psychological Counselling, Faculty of Education Agri Ibrahim Cecen University Ağrı Turkey; ^4^ Department of Psychology, Faculty of Science and Letters Agri Ibrahim Cecen University Ağrı Turkey; ^5^ Department of Social and Educational Sciences Lebanese American University Beirut Lebanon

**Keywords:** 4C, a mixture modeling approach, cognitive flexibility, latent profile analysis, thinking skills

## Abstract

**Objectives:**

Limited research utilized a person‐centered approach in examining 21st‐century skills. This study used latent profile analysis to explore the relationships between resistance to change, cognitive flexibility, and 21st‐century skills, including creativity, cooperativity, critical thinking, communication, and problem‐solving.

**Materials and Methods:**

A convenience sampling approach was used to recruit 502 individuals (342 females; mean_age_ = 21.12 ± 2.34 years) via an online survey.

**Results:**

The results showed that creativity, cooperativity, critical thinking, communication, problem‐solving, and resistance to change were determined as profile indicators. The cognitive flexibility of the subclasses was also predicted. Furthermore, the results revealed four distinct profiles: (i) average level across all skills (5.8%); (ii) low risk for most skills, moderate for cooperativity (18.1%); (iii) high risk for all skills (55.2%); and (iv) moderate risk for most skills, moderate for cooperativity (20.9%).

**Conclusions:**

The study highlights the importance of cognitive flexibility in identifying risk profiles. Using mixture modeling provides a fresh perspective for researchers, potentially aiding in targeted interventions for at‐risk university students to enhance their 21st‐century skills.

## Graphical abstract

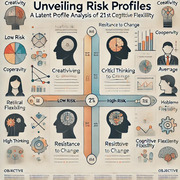

## 1 Introduction

In this century, the demands that individuals face in every field, from education to the business world, are undergoing a radical transformation (Binkley, Erstad, and Herman 2012). Traditional knowledge and skills have become inadequate in the rapidly changing world conditions; new and more complex competencies have gained importance. A lifestyle on the basis of technology requires individuals to develop not only technical skills but also broader capabilities such as critical thinking, problem‐solving, and digital literacy (Tight 2021). In this context, 21st‐century skills play a role in individuals’ ability to keep up with this transformation and actively contribute to society. Consequently, there is a rising apprehension over the disparity in abilities worldwide (Mahmud and Wong 2022). Given the current era's swift technological advancements, globalization, and social transformations, the significance of these competencies is growing for everyone. Therefore, developments and transformations can affect adolescents’ improvement, social relations, and mental health. This exacerbates the challenge of transitioning from childhood to adulthood (Haag, Fantoni, and Dubal 2022). In a globalized society, there is an increasing need for individuals with communication and problem‐solving skills who can adapt to the changing nature of work (Hood and Creed 2019).

## 2 Literature Review

### 2.1 21st‐Century Skills

The classifications of 21st‐century skills vary due to their pervasive influence across all sectors of society (McComas 2014). Van Laar et al. (2020) identified seven skills: “technical, knowledge management, communication, cooperation, creativity, critical thinking and problem‐solving.” Trilling and Fadel (2009) came up with another list of skills known as the “7Cs,” including (i) critical thinking and problem‐solving, (ii) creativity and innovation, (iii) collaboration, teamwork, and leadership, (iv) cross‐cultural understanding, (v) communications, information, and media literacy, (vi) computing and ICT (information and communication technology) literacy, and (vii) career and learning self‐reliance. The partnership for 21st‐century skills emphasizes the pivotal competencies of critical thinking and problem‐solving, communication, collaborative abilities, and the capacity for creativity and innovation, collectively referred to as the “4Cs” (Häkkinen et al. 2017). In a technologically advanced and information‐rich society, critical analysis, synthesis, and evaluation capacity are important for decision‐making and overcoming complex obstacles (Benner, Hughes, and Sutphen 2008). Guilford's approach, which incorporates divergent and convergent thinking and links both to creativity, also reinforces this notion (Jiang et al. 2023). Moreover, creativity fosters individuals to think outside conventional boundaries, produce novel concepts, and tackle challenges from inventive viewpoints (Sun et al. 2022). Proficiency in working efficiently within heterogeneous teams, articulating concepts with clarity, and cooperating across cultural and geographical barriers is crucial for achieving success in professional and social environments (Benishek and Lazzara 2019). Cooperativity encourages the exchange of ideas, stimulates creativity, and supports the development of creative solutions to complex problems (Paulus, Baruah, and Kenworthy 2018). Effective communication is important for building relationships, resolving conflicts, and communicating ideas to other populations (Altman et al. 2023). With globalization, individuals are faced with a multicultural and multilingual atmosphere. These skills help individuals adapt to changing conditions and environments, helping them to be more successful. When those skills are not acquired sufficiently, negative situations such as not being able to work flexibly, not being able to adapt, and not being able to use technological devices sufficiently may occur. For this reason, researchers see the acquisition of these skills as a necessity (Benishek and Lazzara 2019; Jiang et al. 2023). The individual's requirements for the needs of the rapidly changing world include this skill. Moreover, developing these skills (i.e., 4Cs) provides individuals with the ability to produce solutions to issues that have become more complex with technology. As a result, it provides an opportunity for individuals to be more resilient.

### 2.2 Resistance to Change

Change is difficult because it requires great effort. Even those who are willing to do so can feel great anxiety, helplessness, and workload (Rosenberg 2023). The dynamic structure of societies is constantly faced with changes, but these changes are not always welcomed positively by individuals. People can develop resistance to change due to the uncertainty created by the unknown (Mareš 2018). This resistance can manifest itself in various ways, from reluctance to accept new technologies or processes to direct opposition to change (Scholkmann 2021). Individuals keep their distance from innovations due to status, identity, or security concerns. It can increase even more with the fear of affecting their roles or relationships. To overcome resistance to change, individuals need to have sufficient information about the process. Understanding why change is necessary allows people to participate in this process more consciously. Effective leaders reveal the reasons behind the change and can alleviate this resistance by including employees in the processes. Critical thinking, creativity, collaboration, and communication skills come into play during this time.

Critical thinking skills can be used to identify possible risks or opportunities that change may bring more clearly. In addition, the nature of creativity is related to change (Thornhill‐Miller et al. 2023). Therefore, the level of creativity is inversely associated with resistance to change (Simonton 2013). Similarly, cooperation is an important tool for change. In addition, cooperation encourages individuals to prefer change (Lee et al. 2016). All these 4Cs may have a preventive function against resistance to change. On the other hand, acquiring these skills helps individuals to keep up with the rapid change in the 21st century and helps them to evaluate opportunities and use their potential to the fullest.

### 2.3 Cognitive Flexibility

Cognitive flexibility refers to the human ability to adapt to continuously changing environments in response to new situations. It has been associated with various goal‐oriented behaviors, including creativity, problem‐solving, multi‐tasking, and decision‐making. Three important concept characteristics are involved in this definition: First, it is the ability to learn from experience. Second, it implies adapting cognitive processing strategies to novel situations. Third, a strategy is a sequence of operations for searching through a problem space (Payne et al. 1993). Moreover, the multidimensional construct highlights the ability to modify or shift between cognitive sets or strategies in response to environmental changes (Moore and Malinowski 2009). On the basis of the cognitive neuroscience perspective, it has been regarded as a characteristic of cognitive control (i.e., set‐shifting) or the manifestation of multiple cognitive control processes that operate sequentially or in parallel (Zaehringer et al. 2018). Therefore, considering its complex and multidimensional structure, addressing its behavioral dimensions and scale‐based applications in one study is quite challenging. Individuals with such aptitudes ensure their triumph in an ever‐evolving globe.

Individuals break free from rigid thinking and behavior, generate innovative solutions, and find new ideas for complex challenges. Hence, it can be linked to the attributes of creativity and critical thinking (Chen et al. 2014; Orakcı 2021). In addition, adaptability, a critical skill for success in dynamic and unpredictable environments, is also developed through it (Laureiro‐Martínez and Brusoni 2018). Changing individuals’ cognitive patterns is a fundamental aspect of their ability to effectively adjust to changes in their surroundings by overcoming their instinctive responses (Diamond 2013). It enables individuals to thrive in the face of uncertainty by enabling them to see change as an opportunity. Cognitive flexibility also promotes cooperativity and teamwork, one of the 4Cs (Naamati‐Schneider and Alt 2023). It facilitates individuals in navigating diverse viewpoints, solving problems, and discovering shared understanding, eventually resulting in enhanced and fruitful cooperation. It contributes to more efficient and harmonious collaborations. Moreover, cognitive flexibility, which is also closely related to critical thinking, allows individuals to evaluate different options impartially and make conscious decisions (Scheibling‐Sève, Pasquinelli, and Sander 2022). The capacity for flexible thinking, especially in the face of complex problems, facilitates the development of more effective solutions.

### 2.4 Present Study

This study is contextualized according to Guilford's thinking model, complex adaptive systems (CAS), collaborative leadership theory (CLT), and innovation theory frameworks. Guilford's model includes divergent and convergent thinking that links thinking and creativity (Loes and Pascarella 2017). It emphasizes the importance of thinking styles in predicting the potential for creative thinking and problem‐solving. Therefore, developing creativity can provide the innovative approaches needed for problem‐solving. However, according to CAS, individuals’ reluctance to adapt to innovations may lead to resistance to change (Brainard and Hunter 2015). Resistance can prevent the implementation of 21st‐century skills. In addition, according to innovation theory, resistance and cognitive flexibility are related to creativity. Individuals with higher levels of cognitive flexibility are more creative (Kozioł‐Nadolna 2020). It is also an advantage in dealing with resistance to change. In dealing with resistance to change, CLT also emphasizes the importance of communication and cooperation (Shu and Wang 2021). By developing creativity and critical thinking skills, collaborative organizations can provide the innovative approaches needed to solve problems (Andrews‐Todd and Forsyth 2020; Sun et al. 2020). Problem‐solving in the cognitive dimension is also associated with communication, which provides interaction in the social dimension of these frameworks (Graesser et al. 2018).

Beyond the theoretical framework, empirical research shows that critical thinking and creativity benefit undergraduates’ problem‐solving ability. They are also associated with academic achievement (Almulla 2023; Park et al. 2021). Furthermore, collaborative problem‐solving develops creativity and critical thinking skills (Norris, Taylor, and Lummis 2023; Xu, Wang, and Wang 2023). Hon, Bloom, and Crant (2014) highlighted that resistance to change is a significant barrier to creativity. Developing effective communication and cooperation can ensure motivation in the face of resistance (Errida and Lotfi 2021; Khaw et al. 2023). Critical thinking significantly improves performance during change and reduces resistance (Kogetsidis 2012). Di Fabio and Gori (2016) indicated that cognitive flexibility also effectively reduces resistance to change. Cognitive flexibility positively affects collaboration and can reduce resistance to change (Naamati‐Schneider and Alt 2023). It is also associated with higher order thinking skills, such as problem‐solving and critical thinking (Barak and Levenberg 2016). Therefore, it can be effective when coping with change. Similarly, Khalil, Godde, and Karim (2019) stated that the dual model of resistance and cognitive flexibility influences creativity.

This study applied latent profile analysis (LPA) to examine the relationships between creativity, critical thinking and problem‐solving, cooperativity, communication, resistance to change, and cognitive flexibility. One of the main reasons for using LPA is to understand the complexity of these multidimensional 21st‐century skills. Although this method identifies homogeneous subgroups on the basis of individuals’ performance in these skills, it also reveals latent structures and clarifies how skills come together (Spurk et al. 2020). Thus, it also contributes to the creation of development strategies customized to skill levels. Although there are many studies on the 4C skills and their impact on resistance to change and cognitive flexibility among university students, utilizing an LPA may not be enough. In this context, this study aimed to examine the range and intricacy of 21st‐century skills within a sample of the Turkish community. To accomplish this objective, we developed two research questions that directed our investigation: (RQ1): What are the latent profiles of creativity, cooperativity, critical thinking, communication, problem‐solving, and resistance to change? (RQ2): What are the antecedents (i.e., cognitive flexibility, gender, socioeconomic status, and grade level) of the latent profiles of 21st‐century skills?

## 3 Methods

### 3.1 Participants

The sample in this study consisted of 502 individuals (342 females and 160 males). Participants ranged from 18 to 38 years, with a mean age of 21.12 years (SD  =  2.34). A total of 80 (15.9%) participants reported a low self‐expressed socioeconomic level (SESL), whereas 409 (82.5%) reported a moderate SESL, and 13 (2.6%) reported a high SESL. There were 164 (32.7%) freshmen, 97 (19.3%) sophomores, 138 (27.5%) juniors, and 103 (20.5%) seniors among the students. The educational level of the participants’ mothers: 186 (37.1%) of them were illiterate, 201 (40.0%) of them were primary school graduates, 53 (10.6%) of them were secondary school graduates, 44 (8.8%) of them were high school graduates, and 18 (3.6%) had a bachelor's degree. The educational level of the participants’ fathers: 35 (7.0%) were illiterates, 259 (51.6%) of those fathers had primary school graduations, 27 (5.4%) were secondary school graduates, 116 (23.1%) of the participants’ fathers were high school graduates, and 65 (12.9%) of those fathers had bachelor's degrees.

### 3.2 Instruments

#### 3.2.1 Computational Thinking Scale (CTS)

The 29‐item CTS (Korkmaz, Çakir, and Özden 2017) measured individuals’ computational thinking. CTS has five subdimensions (i.e., critical thinking, algorithmic thinking, cooperativity, creativity, and problem‐solving). Items (e.g., “I trust that I will be able to carry out a plan to solve a problem” and “I enjoy cooperative learning experiences with my group members”) are rated on a five‐point Likert scale from 1 (*Never*) to 5 (*Always*). A higher score on the scale indicates an increase in computational thinking. Scores obtained from the scale range from 29 to 145. Cronbach's alpha coefficient of critical thinking, algorithmic thinking, cooperation, creativity, and problem‐solving sub‐dimensions were respectively 0.72, 0.85, 0.87, 0.71, and 0.65. McDonald's *ω*, respectively, were 0.72, 0.86, 0.90, 0.70, and 0.65. The current study's confirmatory factor analysis (CFA) indicated that the model fit the data well: *χ* ^2^/df = 2.75, root means square error of approximation (RMSEA) = 0.06, comparative fit index (CFI) = 0.92, Tucker‐Lewis index (TLI) = 0.91 (Kline 2011).

#### 3.2.2 Communication Skills Assessment Scale (CSAS)

The 25‐item CSAS (Korkut‐Owen and Bugay 2014) assessed individuals’ communication skills. Items (e.g., “In my social interactions, I can accept people as they are” and “Rather than asking specific questions to satisfy my curiosity, I refrain from asking specific questions while listening to the other person”) are rated on five‐point Likert scale from 1 (Never) to 5 (Always). A higher score on the scale indicates an increase in communication skills. For the present study, Cronbach's alpha coefficient was 0.89 and McDonald's *ω* 0.89. Besides, CFA indicated that the model fitted the data well: *χ* ^2^/df = 1.56, RMSEA = 0.03, CFI = 0.98, TLI = 0.98 (Kline 2011).

#### 3.2.3 Resistance to Change Scale (RCS)

The 25‐item CSAS (Oreg et al. 2008) assessed individuals’ communication skills. Items (e.g., “In my social interactions, I can accept people as they are” and “Rather than asking specific questions to satisfy my curiosity, I refrain from asking specific questions while listening to the other person”) are rated on five‐point Likert scale from 1 (Never) to 5 (Always). A higher score on the scale indicates an increase in communication skills. For the present study, Cronbach's alpha coefficient was 0.89 and McDonald's *ω* 0.89. Besides, CFA showed that the model fitted the data well: *χ* ^2^/df = 2.60, RMSEA = 0.06, CFI = 0.92, TLI = 0.90 (Kline 2011).

#### 3.2.4 Cognitive Flexibility Scales (CFS)

The 20‐item CFS (Dennis and Vander Wal 2010; Turkish version: Gülüm and Dağ 2012) assessed individuals’ cognitive flexibility. Items (e.g., “You kept thinking about this incident over and over again, or you were unable to shake it off, which caused you to feel worse and worse” and “Once you started thinking about it, you could not stop”) are rated on five‐point Likert scale from 1 (*Not at all appropriate*) to 5 (*Totally appropriate*). A higher score on the scale indicates an increase in cognitive flexibility. For the present study, Cronbach's alpha coefficient was 0.88 and McDonald's *ω* 0.88. Moreover, CFA showed that the model fitted the data well: *χ* ^2^/df = 2.44, RMSEA = 0.05, CFI = 0.97, TLI = 0.97 (Kline 2011).

### 3.3 Procedure

Participants were selected using convenience sampling. Data were collected using *Google Forms*. The researchers shared the link prepared through social media (e.g., Facebook and Instagram) and instant messaging platforms (e.g., WhatsApp and Telegram) with the participants. The research could continue for those who marked their consent on the first page of the prepared form stating that they volunteered to participate. Detailed information regarding the study duration and objectives was provided. To begin filling out the questionnaires, participants were required to read and sign an informed consent outlining the study's aims, methodologies, and privacy protection standards. The criteria for inclusion in the study were being over 18 years of age, participating voluntarily, and being a university student. Exclusion criteria were not having any clinical diagnosis. Moreover, five items instructed participants (e.g., “Please mark option 4 in this question”) to determine screening question errors. The study did not include participants who failed these questions and had incomplete questionnaires. Participants may also withdraw from the study at any time. They were asked not to write any information indicating their identity to protect the individual's personal information.

### 3.4 Data Analysis

As part of the research process for the analysis and descriptive statistics, assumptions were examined before the analysis. A missing data analysis was performed on the study's data to identify extreme values and missing data. Eight extreme values were determined. The “*z* score” method was used to detect outliers in the data set. Using this technique, individuals with z‐scores greater than +/‐3 standard deviations from the mean were identified as exceeding the threshold value (Yaro, Maly, and Prazak 2023). They were excluded from the data set. Using the remaining 502 data, a further analysis was conducted. A subsequent analysis was conducted to determine whether the data satisfied the normality of distribution of the assumptions, multivariate normality, linearity, and multicollinearity. The analysis was found to be by all assumptions. A CFA was conducted to verify the validity of the measurement instruments. As a result of the fit index being examined, *χ* ^2^ values were significant (*p* > 0.05), and *χ* ^2^/df values were below 5 (Tabachnick and Fidell 2007). The RMSEA and the CFI were used in addition to the TLI and the CFI. RMSEA was cut off at 0.08, CFI was cut off at 0.90, and TLI was cut off at 0.90 (Kline 2011).

A general pattern was established through an analysis of the variables, with *z* scores calculated on the basis of their means and standard deviations. This method allowed us to classify different profile characteristics into categories. Using *z* scores, which measure deviation from the mean in terms of standard deviations, the categories of high, moderate, and low were defined. Specifically, a *z* score between −2 and −3 indicates “high risk,” between −1 and −2 indicates “moderate risk,” and between 0 and −1 indicates “low risk.” This statistical approach is widely used for assessing performance differences within a normally distributed population.

There were two steps in the main statistical analysis. Initially, LPA was performed on creativity, cooperativity, critical thinking, communication, problem‐solving, and resistance to change. An LPA uses continuous variables to compute latent clusters (Muthén and Muthén 1998–2017). First, it was determined how many profiles each individual created on the basis of their group membership. The LPA approach allows researchers to identify homogenous profiles of people within a larger heterogeneous sample. Bayesian information criterion (BIC), adjusted Bayesian information criterion (ABIC), Akaike information criterion (AIC), Lo–Mendell–Rubin likelihood ratio test (LMR‐LRT), bootstrapped likelihood ratio test (BLRT), and entropy values determine the number of latent profiles. Model fit depends on lower BIC and ABIC values. The LMR‐LRT value is used to compare the differences in fit between the models, and a significant LMR *p* value indicates that adding a new profile to the model will improve it. Subsequently, the typical characteristics of the individuals in the latent profiles are described. To assess whether the detected profiles differed on the basis of the variables studied, a one‐way analysis of variance (ANOVA) was conducted. Afterward, a multinomial logistic regression analysis was conducted to examine the relationship between cognitive flexibility and latent classes. Mplus version 7.0 (Muthén and Muthén 1998–2017) was used to analyze latent profiles. Further analyses were performed using the SPSS 26 and JASP version 0.16.1 package program, including ANOVA and multinomial logistic regression.

## 4 Results

### 4.1 Descriptive Statistics

Table 1 illustrates the Pearson correlations among all the main variables in the study (i.e., creativity, cooperativity, critical thinking, problem‐solving, communication, resistance to change, and cognitive flexibility). The results of bivariate correlations indicated that all variables were significantly correlated.

**TABLE 1:** Descriptive statistics and bivariate correlations among variables (*N* = 502).

	1	2	3	4	5	6	7
1. Creativity	—						
2. Cooperativity	0.31^**^	—					
3. Critical thinking	0.49^**^	0.22^**^	—				
4. Problem‐solving	0.34^**^	0.31^**^	0.40^**^	—			
5. Communication	0.51^**^	0.31^**^	0.41^**^	0.37^**^	—		
6. Resistance to change	−0.19^**^	−0.21^**^	−0.20^**^	−0.42^**^	−0.16^**^	—	
7. Cognitive flexibility	0.51^**^	0.25^**^	0.51^**^	0.51^**^	0.61^**^	−0.34^**^	—
Mean	33.57	15.13	18.54	23.02	103.26	49.41	76.51
Std. Deviation	3.84	3.74	3.33	3.89	11.28	9.04	11.12
Skewness	−0.48	−0.88	−0.09	−0.29	−0.39	0.14	−0.04
Kurtosis	0.20	0.44	−0.37	−0.20	0.26	0.44	−0.32

** *p < *0.001.

### 4.2 Latent Profile Analysis

As a first step, a one‐class solution was used for LPA. This was followed by the addition of new profiles to the model. Table 2 shows the class solution between two and six used profiles. The profiles’ number has been increased to 6 to determine the optimal number of profiles. It was determined that four profiles were the most appropriate number of profiles for the data. Table 2 reveals that the BIC, ABIC, and AIC values are within a small range of each other after two profiles. Although the LMR‐LR value of five profiles appears insignificant, the LMR‐LR value of four profiles appears significant. Therefore, the *k* − 1 class number is regarded as the most desirable. For the four‐class model, the entropy value was determined to be 0.81. It was determined that this value was acceptable. An entropy value greater than 0.80 indicates a high degree of differentiation between the classes. Accordingly, after interpreting these results, the 4‐profile solution was deemed the most fitted for the data.

**TABLE 2:** The latent profile analysis (LPA) fit indices on creativity, cooperativity, critical thinking, problem‐solving, communication, and resistance to change.

Model	BIC	ABIC	AIC	LMR‐LRT(*p*)	BLRT	Entropy	*n*
1 Profile	18,506.634	18,468.545	18,456.011	—	—	—	502
2 Profiles	18,155.609	18,095.302	18,075.456	385.694 (0.0043)	−9216.005 (0.0000)	0.650	295, 207
3 Profiles	18,046.270	17,963.744	17,936.586	149.437 (0.0224)	−9018.728 (0.0210)	0.789	65, 108, 329
**4 Profiles**	**18,004.622**	**17,899.878**	**17,865.408**	**83.265 (0.0308)**	**−8942.293** (**0.0284**)	**0.810**	**29, 91, 277, 105**
5 Profiles	18,001.124	17,874.161	17,832.380	45.972 (0.2946)	−8899.704 (0.2864)	0.826	58, 106, 225, 80, 33
6 Profiles	18,007.192	17,858.010	17,808.918	36.621 (0.2639)	−8876.190 (0.2574)	0.779	53, 99, 48, 94, 175, 33

*Note*: Optimal model in bold.

Abbreviations: ABIC, Adjusted Bayesian information criterion; AIC, Akaike information criterion; BIC, Bayesian information criterion; BLRT, bootstrapped likelihood ratio test; LMR‐LRT, Lo–Mendell–Rubin likelihood ratio test.

**FIGURE 1:**
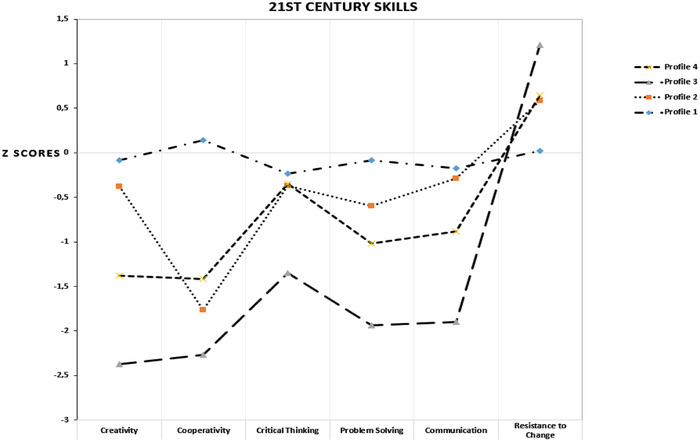
Line graph regarding studying variables and latent profiles.

A general pattern was then established by analyzing the variables. *z* scores were calculated on the basis of the variables’ means and standard deviations. Figure 1 illustrates the different characteristics of the profiles. The students in Profile 1 have average creativity, cooperativity, critical thinking, problem‐solving, communication, and resistance to change. There are 29 students in this group (5.8% of the participants). Students in Profile 2 demonstrate a low risk for creativity, critical thinking, problem‐solving, and communication and a moderate risk for cooperativity. There are 29 students in this group (18.1% of the participants). Students in Profile 3 demonstrate a high risk for creativity, cooperativity, critical thinking, problem‐solving, and communication. They also have the highest level of resistance to change. Profile 3 comprised 277 students (55.2% of the sample). Students in Profile 4 show moderate risk for creativity, critical thinking, problem‐solving, and communication and moderate risk for cooperativity. A total of 105 students are included in Profile 4 (20.9% of the participants). There was a similar level of resistance to change in Profiles 3 and 4.

The latent profiles of creativity, cooperativity, critical thinking, problem‐solving, communication, and resistance to change were compared using one‐way ANOVA after determining the students’ optimal number of latent profiles. According to Table 3, all latent profiles have significant *F* values.

**TABLE 3:** Analysis of variance (ANOVA) results examined the differences across the four latent profiles.

Variables	Profile 1 (*n *= 29) M(SD)	Profile 2 (*n *= 91) M(SD)	Profile 3 (*n *= 277) M(SD)	Profile 4 (*n *= 105) M(SD)	*F* (3, 498) *η* ^2^	Post hoc Bonferroni comparison
Critical thinking	14.79 (2.21)	22.13 (2.37)	18.76 (2.57)	15.86 (2.63)	122, 31^***^ (0.14)	2 > 1,3,4; 3 > 1,4
Cooperativity	12.79 (3.56)	18.07 (2.90)	14.89 (3.45)	13.86 (3.78)	31, 91^***^ (0.10)	2 > 3 > 1; 2 > 4
Creativity	25.69 (2.65)	37.37 (2.37)	33.9 (2.71)	31.58 (3.27)	154, 39^***^ (0.28)	2 > 3 > 4 > 1
Communication	82.34 (8.47)	116 (6.31)	103.92 (8.09)	96.29 (8.44)	174, 60^***^ (0.37)	2 > 3 > 4 > 1
Problem‐solving	18.83 (2.85)	26.73 (3.01)	23.53 (2.88)	19.61 (3.42)	111, 46^***^ (0.14)	2 > 1,3,4; 3 > 1,4
Resistance to change	53.55 (8.53)	44 (9.85)	49.33 (8.24)	53.15 (8.12)	21, 16^***^ (0.12)	4 > 2,3; 1,4,3 > 2

*** *p* < 0 .001.

In the final stage of the analysis, multinomial logistic regression analysis was utilized using descriptive variables and cognitive flexibility to predict profile membership. First, we compared the likelihood of membership in Profile 2 versus Profile 1 (reference group). As shown in Table 4, we detected significant main effects for being female (OR = 6.56, *p* < 0.001) and cognitive flexibility (OR = 2.33, *p* < 0.001). Results showed that a comparison of Profile 3 versus Profile 1 showed that being female (OR = 1.71, *p* < 0.001) and cognitive flexibility (OR = 3.19, *p* < 0.001) were significant predictors. Lastly, a comparison of Profile 4 versus Profile 1 showed that being female (OR = 3.32, *p* < 0.001) and cognitive flexibility (OR = 1.23, *p* < 0.001) were significant predictors. These findings reveal that a 1‐unit increase in these dimensions is associated with a 1‐unit increase in the chances of belonging to those profiles.

**TABLE 4:** Multinomial logistic regression results predicting profile membership.

	Profile 2 vs. Profile 1		Profile 3 vs. Profile 1		Profile 4 vs. Profile 1	
	OR	95% CI	OR	95% CI	OR	95% CI
*Gender^1^*						
Female	6.56^**^	[1.54–28.03]	1.71^***^	[1.52–1.93]	3.32^*^	[1.15–9.55]
*Socioeconomic status (SES)^2^*						
Low SES	0.81	[0.10–37.52]	0.03	[0.034–39.25]	0.17	[0.17–104.42]
Moderate SES	0.68	[0.11–19.74]	0.04	[0.044–25.96]	0.16	[0.16–59.85]
*Grade level^3^*						
Freshmen	2.46	[0.37–16.27]	0.44	[0.444–10.40]	0.69	[0.69–10.47]
Sophomores	4.93	[0.69–35.34]	0.70	[0.706–16.60]	0.84	[0.84–11.08]
Juniors	7.49	[0.98–57.32]	0.48	[0.483–14.81]	1.08	[1.08–22.55]
Cognitive flexibility	2.33^***^	[2.03–2.68]	3.19^***^	[0.95–10.74]	1.23^***^	[1.11–1.35]

*Note*: 1 Reference group = Male; 2 Reference group = High SES; 3 Reference group = Senior.

* *p*  <  0.05

** *p * < 0 .01

*** *p * < 0 .001

## 5 Discussion

Individuals’ behaviors in the change process are closely linked to their cognitive and social skills, which may lead to resistance to change. In this study, individuals’ levels of resistance to change and their creativity, cooperativity, critical thinking, and problem‐solving skills, known as 4Cs, are discussed. The study examines the latent model between these skills and resistance to change. It adopts a person‐centered approach by going beyond traditional analyses and focusing on the latent structures of individuals with different profiles. This approach reveals how individuals’ cognitive and 4C skills differ in the face of change and how resilience levels are formed according to these profiles. However, these results are based on self‐report scales. In particular, subjective measurements of constructs such as cognitive flexibility and creativity may not fully reflect the actual dynamics of these constructs (Kupis et al. 2021; Wahbeh et al. 2024). Indeed, cognitive flexibility is a complex structure that is difficult to measure. This structure can be measured with neuropsychological, self‐report, and neuroscientific approaches (Hohl and Dolcos 2024). Similarly, creativity consists of the dimensions of novelty, relevance, and impact and can be measured indirectly (e.g., self‐report questionnaires). In this study, cognitive flexibility and creativity were measured on the basis of self‐report.

The results showed that a model consisting of four profiles was most appropriate. These profiles explain resistance to change by reflecting combinations of the 4Cs. The fact that the study sample had different characteristics made it possible to analyze these relationships. The latent structure of Profile 1 indicated that participants had the lowest resistance to change and, in turn, showed the highest levels of creativity, cooperativity, critical thinking, problem‐solving, and communication. It indicates a strong link between high skill levels and low resistance to change. Profile 2 had moderate resistance to change, creativity, critical thinking, problem‐solving, communication, and low cooperativity. The profile reveals high performance in some skills (i.e., creativity, critical thinking, and communication) and moderate resistance to change. Profile 4 had moderate resistance to change, cooperativity, critical thinking, low creativity, problem‐solving, and communication. Finally, Profile 3 had the highest resistance to change and the lowest levels of creativity, cooperativity, critical thinking, problem‐solving, and communication. It also indicates low performance in all skill areas with the highest resistance to change.

The profiles reveal the interrelationship between 4Cs and resistance to change. In this study, 4Cs were inversely related to resistance to change; however, this relationship differed according to the latent profiles to which individuals belonged. This inverse proportion was especially evident in Profiles 1 and 3, whereas resistance to change levels remained the same in Profiles 2 and 4, and differences were observed in other variables. The results show that the concept of resistance to change is influenced by the individual's unique characteristics and abilities (Kim and Park 2023). For example, Sucuoğlu, Sarıkaya, and Bahçelerli (2022) found that school administrators’ adaptation to change is related to 21st‐century skills. Çavuş and Helvaci (2021) reported that the competencies of teachers and school administrators to use 21st‐century skills and their level of readiness for change are at a high level. The study's findings extend the literature's results on the relationship between resistance to change and 21st‐century skills. It shows that these skills and their relationship with resistance to change may differ. Innovation Theory emphasizes the role of creativity in managing change and shows that creative thinking skills will differ from person to person (Diala and Ude 2015). The climate of modernity, empowering leadership, and supportive coworkers can reduce the harmful effects of resistance to change, which is one of the barriers to creativity (Hon, Bloom, and Crant 2014). In education and business, focusing on developing creativity and critical thinking skills can effectively deal with resistance to change. Studies also emphasize the role of effective communication in reducing resistance to change (Cheraghi et al. 2023; Li 2024). Simões and Esposito (2014) reported that communication is effective in dealing with resistance to change. Collaborative leadership and communication play an important role in determining the readiness of profiles for change (Paulus, Baruah, and Kenworthy 2018; Elgohary and Abdelazyz 2020). Moreover, CLT also emphasizes the impact of cooperativity on change readiness (Jiang 2023; Shu and Wang 2021). A collaborative teamwork culture is effective in guiding and preparing for change (Ellis et al. 2023). Collaborative leadership approaches and effective communication strategies can play a key role in the success of organizations in change processes. Guilford's convergent thinking model explains the role of critical thinking, creativity, and problem‐solving skills in resistance to change. In addition, it becomes more explicit how these skills interact in different profiles of individuals (Drago and Heilman 2012). Twenty‐first–century skills can be effective in coping with resistance to change (Tangney et al. 2023; Warrick 2023). As a result, individuals’ adaptation to change processes is related to their profiles. In the process of change, the cognitive characteristics of individuals, along with their skills, are also effective. Cognitive flexibility is an important skill that reduces resistance to change according to differences between profiles and is negatively related to resistance to change (Chung, Su, and Su 2012). Similarly, there is a relationship between cognitive flexibility and resilience (Nakhostin‐Khayyat et al. 2024). Higher levels of cognitive flexibility can encourage people to use a variety of ways of thinking to promote creativity (Chen et al. 2019). Furthermore, individuals with high cognitive flexibility can think critically and provide creative solutions (Laureiro‐Martínez and Brusoni 2018). Consequently, the profiles that emerged are consistent with the other research results.

Findings from multinomial logistic regression highlight the role of cognitive flexibility in predicting latent profiles in the context of 21st‐century skills. They indicated that female and cognitive flexibility were antecedent variables of latent profiles. Consistent with the literature, socioeconomic status and grade level have no predictive effect on latent profiles (Karakuş 2024; Schrempft et al. 2022). Yu, Zhao, and Hou (2023) reported that cognitive flexibility has a positive effect on entrepreneurial creativity. In relation to creativity, reflective thinking is a positive, significant predictor of cognitive flexibility (Orakcı 2021). Ouyang, Liu, and Gui (2023) explained the combined effect of cooperation and competition on creativity through changes in cognitive flexibility. Furthermore, communication within the family is related to the cognitive flexibility of young adults (Koesten et al. 2009). The results show that the 4Cs and change resistance skills profiles are related to cognitive flexibility and female gender. Consistent with the literature, the results of this study revealed that female gender and cognitive flexibility significantly influenced the profiles. It provides important insights into the impact of gender and cognitive flexibility on individuals’ resistance to change and 21st‐century skills. The effect of female gender on the profiles contributes to the debate in the literature on how gender roles can contribute to 4Cs (Saad et al. 2024). In addition, individuals’ limited opportunities for cognitive and social development may explain why socioeconomic status and grade level have no effect on the profiles, which might lead to the effect of these constructs not being observed (Acar et al. 2023). It may also be associated with the lack of enriching environmental experiences necessary for the development of cognitive flexibility (Clearfield and Niman 2012).

### 5.1 Limitations and Recommendations

The study has several limitations that should be considered. The study was conducted using data based on self‐report scales. Participants’ self‐assessment of their skills may lead to biases (e.g., social acceptability), making it difficult to measure complex constructs (e.g., cognitive flexibility and creativity). We acknowledge that cognitive flexibility needs to be assessed using behavioral approaches and experimental methods. Therefore, it is necessary to consider that results based on subjective evaluations do not fully reflect the actual performance of individuals. Although the data were from a relatively large cohort, the sample consisted of university students. In addition, the cross‐sectional study limits the ability to establish cause‐and‐effect relationships. Therefore, future longitudinal studies are needed to examine the observed dynamic relationships more deeply.

The generalizability of the findings to other age groups or professional contexts may also be limited by the focus on university students only. It is suggested that further research expand the participant base to increase demographic diversity and demonstrate how these cognitive skills and levels of resistance to change manifest in different populations. The present study provides important findings regarding the relationships between cognitive flexibility, 4Cs, and resistance to change. Considering the limitations, it is suggested that further studies based on more objective measurements and with more diverse sample groups will make significant contributions to the literature in this field. Further research could explore the underlying mechanisms and possible mediating or moderating factors that may influence these relationships. The current study is limited to only four 21st‐century skills (i.e., “4Cs”), so further research might include other skills to compare and contrast the results.

## 6 Conclusion

The research presented a thorough analysis of the latent model of creativity, cooperativity, critical thinking, problem‐solving, communication, and resistance to change. Each profile demonstrated an interaction between the 4Cs and resistance to change. The four different profiles identified revealed the complex relationships between these elements. In particular, the inverse relationship between 4Cs and resistance to change across all profiles highlights the important role of creativity, critical thinking, and problem‐solving in influencing adaptive responses during periods of change. This study is consistent with the existing literature, making links with Innovation Theory and Guilford's model, and further emphasizes the integrated nature of divergent and convergent thinking in effective problem‐solving. The collaborative aspects of cooperation and communication emerge as key components in reducing resistance to change and fostering an environment conducive to innovation. As organizations grapple with the complexities of change, these insights provide a valuable basis for tailored interventions and strategies to foster a culture of adaptation, collaboration, and successful organizational transformation.

## Author Contributions

Study conception/design: Alican Kaya, Suat Kaya, and Muhammed Celal Uras. Data collection: Alican Kaya, Suat Kaya, and Muhammed Celal Uras. Analysis: Alican Kaya. Drafting of the manuscript: Alican Kaya, Suat Kaya, and Muhammed Celal Uras. Statistical expertise: Alican Kaya. Administrative/technical/material support: Alican Kaya, Suat Kaya, and Muhammed Celal Uras. Writing‐review and editing: Murat Yildirim. All authors read and approved the final manuscript.

## Ethics Statement

The ethical approval for this study was also obtained from the ethics committee of Agri Ibrahim Cecen University (reference no: 248). The procedures were also carried out in accordance with the Declaration of Helsinki.

## Consent

Consent was obtained from all participants included in the study.

## Conflicts of Interest

The authors declare no conflicts of interest.

### Peer Review

The peer review history for this article is available at https://publons.com/publon/10.1002/brb3.70167.

## Data Availability

The data sets generated during and analyzed during the current study are available from the corresponding author upon reasonable request.
